# Neutrophils as Trojan Horse Vehicles for *Brucella abortus* Macrophage Infection

**DOI:** 10.3389/fimmu.2019.01012

**Published:** 2019-05-07

**Authors:** Cristina Gutiérrez-Jiménez, Ricardo Mora-Cartín, Pamela Altamirano-Silva, Carlos Chacón-Díaz, Esteban Chaves-Olarte, Edgardo Moreno, Elías Barquero-Calvo

**Affiliations:** ^1^Programa de Investigación en Enfermedades Tropicales, Escuela de Medicina Veterinaria, Universidad Nacional, Heredia, Costa Rica; ^2^Centro de Investigación en Enfermedades Tropicales, Facultad de Microbiología, Universidad de Costa Rica, San José, Costa Rica

**Keywords:** *Brucella*, neutrophils, macrophages, Trojan horse, phosphatidylserine

## Abstract

*Brucella abortus* is a stealthy intracellular bacterial pathogen of animals and humans. This bacterium promotes the premature cell death of neutrophils (PMN) and resists the killing action of these leukocytes. *B. abortus*-infected PMNs presented phosphatidylserine (PS) as “eat me” signal on the cell surface. This signal promoted direct contacts between PMNs and macrophages (Mϕs) and favored the phagocytosis of the infected dying PMNs. Once inside Mϕs, *B. abortus* replicated within Mϕs at significantly higher numbers than when Mϕs were infected with bacteria alone. The high levels of the regulatory IL-10 and the lower levels of proinflammatory TNF-α released by the *B. abortus*-PMN infected Mϕs, at the initial stages of the infection, suggested a non-phlogistic phagocytosis mechanism. Thereafter, the levels of proinflammatory cytokines increased in the *B. abortus*-PMN-infected Mϕs. Still, the efficient bacterial replication proceeded, regardless of the cytokine levels and Mϕ type. Blockage of PS with Annexin V on the surface of *B. abortus*-infected PMNs hindered their contact with Mϕs and hampered the association, internalization, and replication of *B. abortus* within these cells. We propose that *B. abortus* infected PMNs serve as “Trojan horse” vehicles for the efficient dispersion and replication of the bacterium within the host.

## Introduction

Polymorphonuclear neutrophils (PMNs) are the first line of defense of the innate immune system against bacterial pathogens (1–3). Upon contacts with invading bacteria, PMNs activate their killing mechanisms, release cytokines, and may generate PMN extracellular traps (3–5). Although PMNs kill most of the microorganisms they interact with, there are some pathogens capable to resist the microbicidal actions of these leucocytes (6).

*Brucella abortus* is a Gram-negative bacteria that cause disease in bovines and humans (7). After host invasion, PMNs are the first immune cells to encounter and phagocytize *Brucella* organisms (8, 9). However, *Brucella*-infected-PMNs release negligible amounts of proinflammatory cytokines, generate low levels of reactive oxygen species and seldom show degranulation (10–12). Moreover, *Brucella* pathogens survive inside PMNs for a protracted period of time (10) and induce the premature death of these cells (12, 13). Although the dying *Brucella*-infected PMNs display phosphatidylserine (PS) on the cell surface, they do not show chromatin condensation or signs of necrosis or oncosis (12). Nevertheless, the exposure of PS on the *B. abortus*-infected PMNs resembles that of apoptotic PMNs. As demonstrated (14), non-infected apoptotic PMNs presenting PS on the surface are removed by macrophages (Mϕs) in a non-phlogistic manner (14). Indeed, the removal of apoptotic PMNs is first established by the release of “find me” signals required for recruitment of mononuclear phagocytes. Then, the recognition of PS on the surface of the apoptotic PMNs constitutes an “eat me” signal, which in course induces the regulated suppression of Mϕs activating mechanisms (14, 15).

We have proposed that the premature PMN cell death induced by *Brucella* organisms may promote the selective non-phlogistic removal of these infected cells by the mononuclear phagocytic system (12, 13). In course, *Brucella* infected PMNs may serve as “Trojan horse” vehicles for efficient bacterial dispersion, intracellular replication and establishing chronic infections, as suggested for other pathogens (16). Here we demonstrate that *Brucell*a-infected PMNs are readily phagocyted by murine Mϕs in a non-phlogistic manner, and that bacteria delivered through PMNs, extensively replicate inside Mϕs. The experiments shown here, are a proof of concept for the “Trojan horse” proposal, which states that *Brucella*-infected PMNs serve as vehicles for Mϕ infection and subsequent dispersion throughout the organism.

## Materials and Methods

### Bacteria and Mouse Strains

*B. abortus* 2308 expressing constitutive red fluorescent protein from *Discosoma coral* (*B. abortus*-RFP), provided by Jean-Jacques Letesson (Unité de Recherche en Biologie Moléculaire, Facultés Universitaires Notre-Dame de la Paix, Namur, Belgium), was used in all experiments. Female BALB/c mice (18–21 g) were supplied by the Escuela de Medicina Veterinaria, Universidad Nacional, Costa Rica, and Instituto Clodomiro Picado, Universidad de Costa Rica, Costa Rica.

### Ethics

Bone marrow (BM) was obtained from mice following the consent and guidelines established by the “Comité Institucional para el Cuido y Uso de los Animales de la Universidad de Costa Rica” (CICUA- 47-12) and in accordance with the corresponding law, Ley de Bienestar de los Animales, of Costa Rica (law 9458 on animal welfare). All animals were kept in cages with food and water *ad libitum* under biosafety containment conditions.

### Infection Protocols

PMNs were obtained from BM and infected *ex vivo* in the presence of anti-*Brucella* antibodies, following previous protocols (13, 17). Briefly, BM cells were isolated from tibia and femur of mice by flushing bones with HBSS (no calcium, no magnesium) or RPMI medium. Then, BM cells were infected with *B. abortus*-RFP (MOI 50) at 37°C for 1.5 h, washed with PBS, suspended in HBSS, and examined by fluorescent microscopy. The composition and proportion of the infected BM cells have been determined in previous work (17). Under the fluorescent microscope, the estimation of infected murine PMNs is a straightforward process due to the unique donut shape of their nuclei. The proportion of infected and non-infected cells were counted by following a meaningful statistical sampling method (18). *B. abortus* PMN infections were confirmed by flow cytometry using *B. abortus*-RFP and PE anti-Ly6G (RB6-8C5) from eBioscience as previously described (17).

Peritoneal Mϕs were harvested and cultured as previously described (19). *B. abortus*-RFP infection (MOI 50) of 2 × 10^5^ RAW 264.7 or peritoneal Mϕ monolayers was performed by using the gentamicin protection assay to avoid extracellular bacteria (20). Additionally, RAW 264.7 or peritoneal Mϕs were infected by co-cultivating with *B. abortus-*infected PMNs as follows. *B. abortus-*infected PMN were washed with PBS to remove extracellular bacteria. Then, *B. abortus*-infected PMNs were suspended in DMEM without gentamicin and added to the Mϕ monolayers at a rate of 1:1 and incubated for one hour at 37°C. After this period, gentamicin was added. Then, cells were cultivated for up to 48 h and CFU counts determined at 3, 7, 24-, and 48-h post-infection. Alternatively, *B. abortus-*infected PMN were pre-treated with 5 μg/cell of Annexin V (Invitrogen) for 15 min (15) before co-cultivation with RAW 264.7 cells. The CFU counts within *B. abortus-*infected PMN added to RAW 264.7 and peritoneal Mϕs monolayers were calculated retroactively by lysing the PMNs and counting bacteria in agar plates. Controls of co-cultivated non-infected PMN with Mϕ monolayers (at rate 1:1) were run in parallel.

### Immunofluorescence

The percentage of cell association (direct cell-cell contact) between *B. abortus-*infected PMN and non-infected PMNs with Mϕs was estimated by fluorescent microscopy at different time points. Infected and non-infected PMNs were fixed with 3.5% paraformaldehyde, centrifuged in a Cytospin 2 (Shandon), mounted with ProLong Gold Antifade reagent with DAPI (Thermo Fisher Scientific), and observed under the fluorescence microscope (Nikon ECLIPSE 80i). Mϕ monolayers co-infected with *B. abortus-*infected PMN were stained with DAPI and FITC-phalloidin (Sigma), fixed and mounted with MOWIOL for analysis as described (12). Controls of non-infected PMNs were used along with the corresponding assays. At least 200 PMNs were counted per slide. Cells were photographed under the fluorescence microscope (Nikon ECLIPSE 80i) using the appropriate color filter channel. Images were cut from microscope field, contrasted and saturated using Hue tool to obtain suitable color separation. Images were merged using Adobe Photoshop 8 software. Internalization of *B. abortus-*infected PMN and non-infected PMNs was documented by live-imaging using Cytation 5 Cell Imaging reader.

### Cytokine Determination

For the quantitative determination of TNF-α and IL-10, the supernatants of the infected Mϕs monolayers were collected at different time points and the concentration of cytokines measured by ELISA according to the manufacturer's specifications (Invitrogen).

### PMNs Cell Death Determination

For cell death analysis, PMNs were stained with Alexa Flour 488 Annexin V (Invitrogen) and PE anti-Ly6G (RB6-8C5) and APC Cy7 anti-CD16/32 antibodies (from eBioscience and BD Bioscience respectively). *B. abortus-*infected PMN cells were analyzed by flow cytometry using a Guava easyCyte (Millipore) and data analyzed using the FlowJo software, version 10.0.7 (Tree Star, Inc.) (13, 21). Evaluation of PMNs cell death assay was carried out as described before (13). Briefly, aliquots of BM were mixed with *B. abortus*-RFP (MOI 50), supplemented with anti-*Brucella* murine serum for opsonization, and incubated under mild agitation at 37°C for up to 4 h. Cells were then suspended in Annexin-binding buffer (Invitrogen) and Annexin V added and incubated for 30 min on ice in the dark. Cells were washed with ice-cold PBS, fixed with 3.2% paraformaldehyde and subjected to flow cytometry analysis.

### Statistics

The Wilcoxon signed-rank test was used to compare the proportion of association between non-infected PMNs and *Brucella*-infected PMNs to Mϕs. Analysis of covariance (ANCOVA) was used to determine the effects of time and treatments on statistical the Log_10_ CFU. Two-way analysis of variance (ANOVA) was used to measure the effect of time and treatments on the percentage of Mϕ infection. Shapiro-Wilks test was applied to assess the normal distribution of data obtained in each experiment, and the Kolmogorov-Smirnov test was applied to data that did not adjust to normality. JMP (https://www.jmp.com) and GraphPad Prism software (https://www.graphpad.com) was used for statistical analysis. Data were processed in Microsoft Office Excel 2016 and GraphPad Prism software. For a meaningful counting number of infected cells, a probability index was followed, according to the total number of PMNs and infected PMNs (18).

## Results

**Figure 1:**
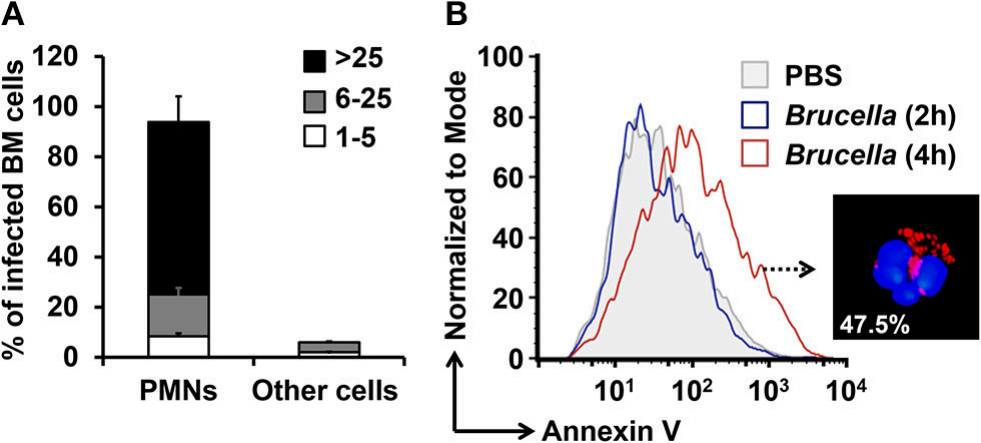
*B. abortus* infect PMNs and promote the exposure of phosphatidylserine. BM cells were incubated with *B. abortus*-RFP (MOI 50) for 4 h. **(A)** Cells were mounted using Prolong Gold containing DAPI (blue nuclei). At least 200 PMNs were counted per sample. The percentage of infection and the number of intracellular bacteria (1–5, 6–25, or >25) per cell was determined by fluorescence microscopy. Cell infections were confirmed by flow cytometry. **(B)** The PMN population analyzed by flow cytometry was gated using anti-Ly6G as PMNs cell marker and analyzed by Annexin V as a cell death marker. These experiments were repeated at least three times.

The limited volume of mouse blood and the low number of PMNs in this fluid, preclude the isolation of a sizeable number of these leukocytes for functional studies. In addition, the extensive manipulation during purification procedures accelerates the cell death of PMNs. In contrast, the number of PMNs in the BM is rather high, comprising between 40 and 50% of all nucleated cells (22). In agreement with previous results (17), close to 94% of the *ex vivo B. abortus* BM-infected cells corresponded to PMNs (Figure 1A). We have previously shown that the remaining infected cells are monocytes or progenitor stem cells (17). The distinction between mononuclear infected cells and infected PMNs is straightforward due to the donut shape of the nuclei of the latter cells. Following this, we then tested if *B. abortus* were capable to induce the premature cell death of BM PMNs, as shown before for blood PMNs (13), up to 47.5% of the *B. abortus*-infected PMN were positive for Annexin V at 4 h post-infection (Figure 1B).

**Figure 2:**
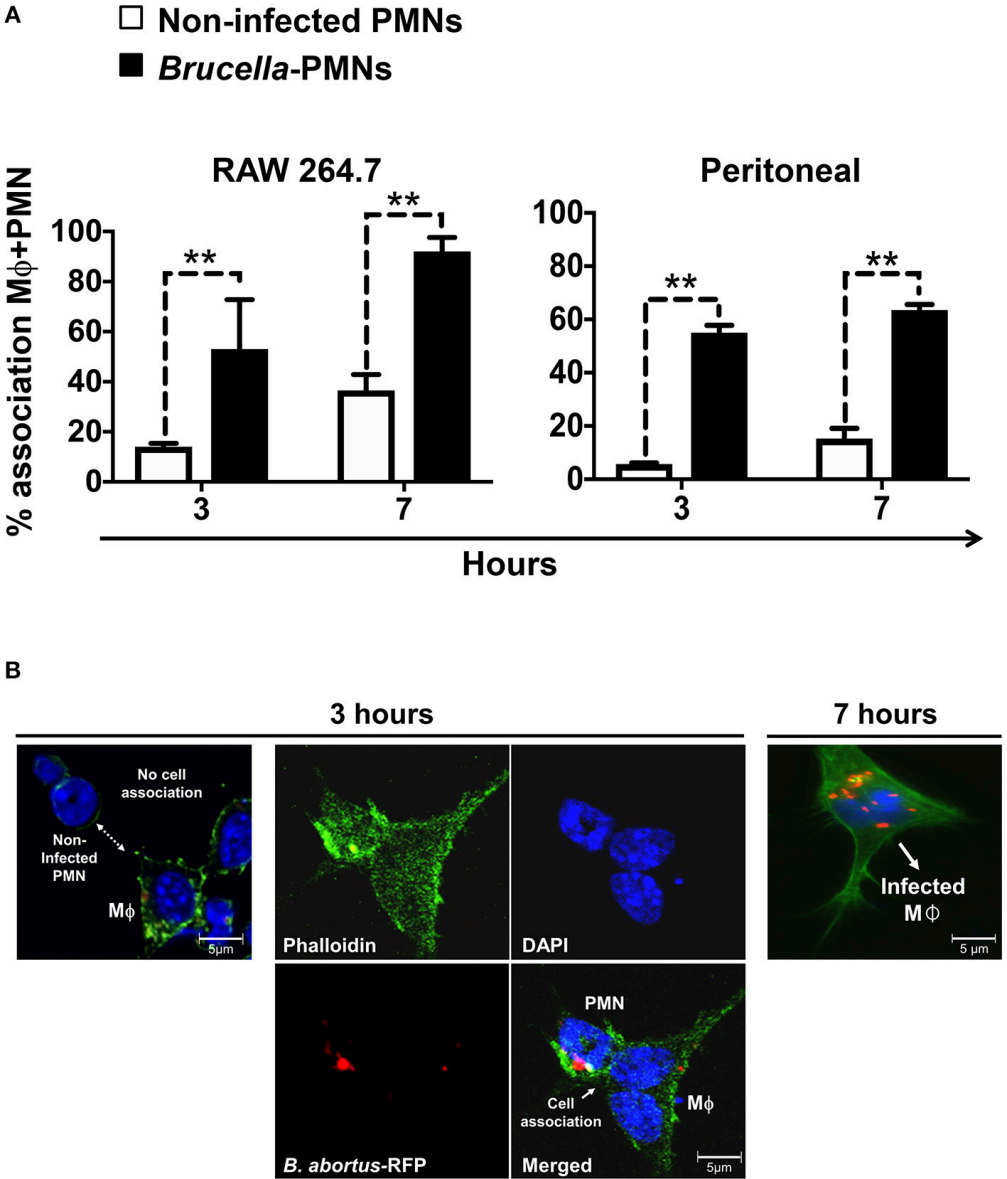
*B. abortus*-infection increase the association of PMN with Mϕs. **(A)** Non-infected or *B. abortus*-RFP-infected PMN were incubated with RAW or peritoneal Mϕs (1:1) at different time points and PMN-Mϕ interactions were quantified. Cells were stained (DAPI, for nuclei; phalloidin-FITC for actin filaments), fixed and mounted with MOWIOL. At least 200 PMNs were counted and the percentage of PMN-Mϕ cell association determined. Values of *p* < 0.01 (**) are indicated in relation to Mϕs incubated with non-infected PMNs. **(B)** RAW Mϕ in the process of association and ingestion *B. abortus*-infected PMN. Infected PMNs are distinguished from other cells by the “donut” shape of their nuclei. Images were photographed under the microscope using the appropriate color filter channel. These experiments were repeated at least three times.

**Figure 3:**
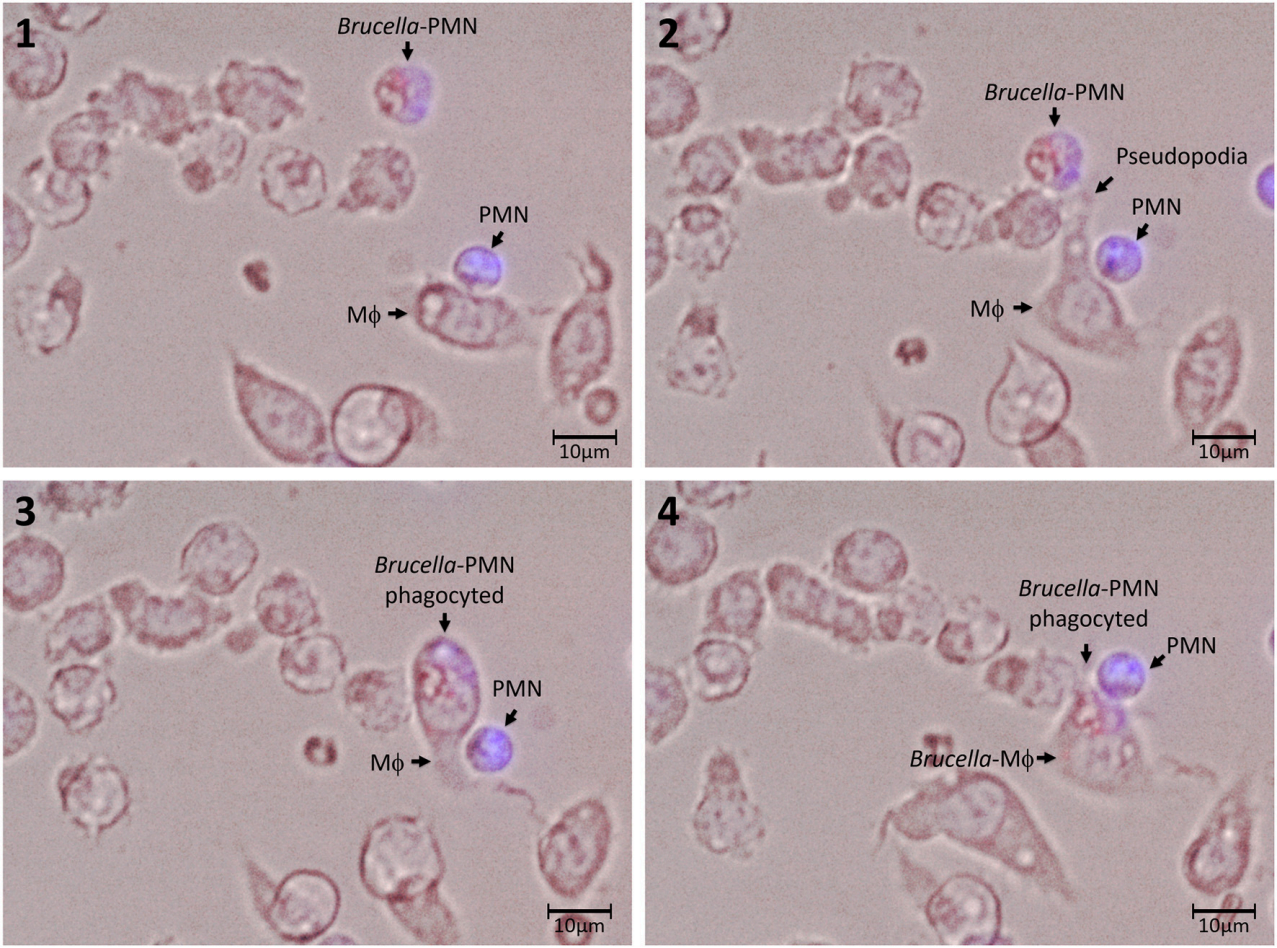
Association and uptake of *B. abortus*-infected PMN by Mϕs. PMNs were incubated with *B. abortus*-RFP (red) (MOI of 50) for 1.5 h; then, cells were pelleted and washed with PBS to remove extracellular bacteria. *Brucella-*infected-PMNs were suspended in DMEM without gentamicin and added to RAW Mϕs monolayers (5 × 10^3^) at a rate of 1:1 and incubated for 10 min at 37°C. After this period, the infected Mϕ monolayers were washed and suspended in DMEM and incubated for up to 5 h. Infected PMNs were stained with Hoescht (blue). Cells were photographed and analyzed under Cytation 3 Cell Imaging Multi-Mode Reader (BioTek) using the appropriate color filter channel. Numbers 1 to 4 correspond to the order in the which images were capture very every 20 min. These experiments were repeated at least three times.

Then, we explored the association of *Brucella*-PMNs to Mϕs by co-cultivating these two cells *in vitro*. As compared to the non-infected PMN controls, a higher proportion of *Brucella-*infected-PMNs associated with RAW and peritoneal Mϕs was detected (Figure 2A). Thereafter, the association between *Brucella-*infected-PMNs and Mϕs, led to the infection of the latter (Figure 2B). This phenomenon was completed before 7 h and was specific since non-infected PMNs were not phagocytized by Mϕs (Figure 3). However, a strict kinetic analysis was precluded, since Mϕ phagocytosis and the concomitant digestion of PMNs was very fast an uneven event over time.

**Figure 4:**
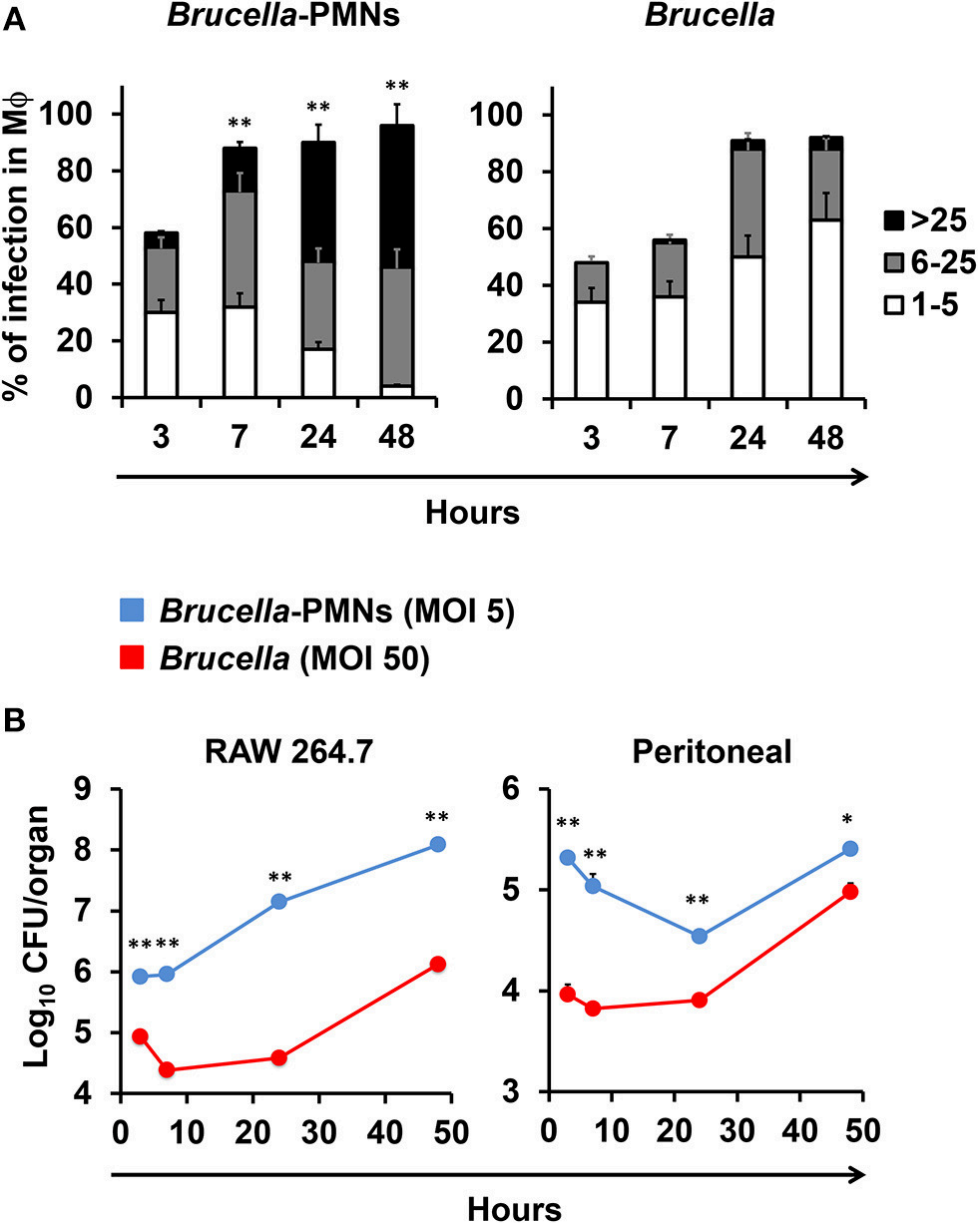
*B. abortus-*infected PMN promote *Brucella* infection of Mϕs. **(A)** RAW Mϕs were incubated with *B. abortus*-RFP alone or with *B. abortus-*infected PMN, then cells were stained (DAPI, for nuclei; phalloidin-FITC for actin filaments), fixed and mounted with MOWIOL, and the number intracellular bacteria (1–5, 6–25, or >25) per Mϕ was determined. The proportion of infection and the number of intracellular bacteria was determined by fluorescence microscopy. At least 200 cells were counted per sample. Values of *p* < 0.05 (*) and *p* < 0.01 (**) are indicated in relation to Mϕs incubated with non-infected PMNs. **(B)** RAW and peritoneal Mϕs were infected with *B. abortus-*RFP alone (MOI 50) or with *B. abortus*-RFP infected PMNs (MOI 5) and CFU determined at different time points. These experiments were repeated at least three times.

Then, we tested the rate of bacterial replication after internalization of Mϕs by *Brucella-*infected-PMNs at 1 and 48 h post-infection. As shown in Figure 4A, *B. abortus* organisms infected Mϕs at higher rates through phagocytosis of *Brucella-*infected PMNs than when infected with bacteria alone. Moreover, the higher efficiency of Mϕ bacterial infection mediated by *Brucella-*infected-PMNs was evident by the different MOIs delivered in each case. Indeed, in the case of *Brucella-*infected PMNs the number of delivered bacteria corresponded to an MOI of 5; that is, ten times lower than the MOI of 50 used to infect Mϕs with bacteria alone. The efficient internalization process promoted higher kinetics of *B. abortus* replication in Mϕs incubated with *Brucella-*infected-PMNs (Figure 4B). In spite of this, the kinetics between RAW and peritoneal Mϕs were different. For instance, RAW Mϕs infected with *B. abortus* alone displayed an initial decline in CFUs at early times of infection, a phenomenon that has been reported before (23). However, after infection of these cells with *Brucella-*infected-PMNs, the initial decline was unnoticeable in these Mϕs; instead, a steady increase in the number of CFUs was observed. In contrast, the kinetic profiles were similar in both, the *Brucella*-PMN infected peritoneal Mϕs and in the controls; though, the number of CFU was always higher in the former infected cells.

**Figure 5:**
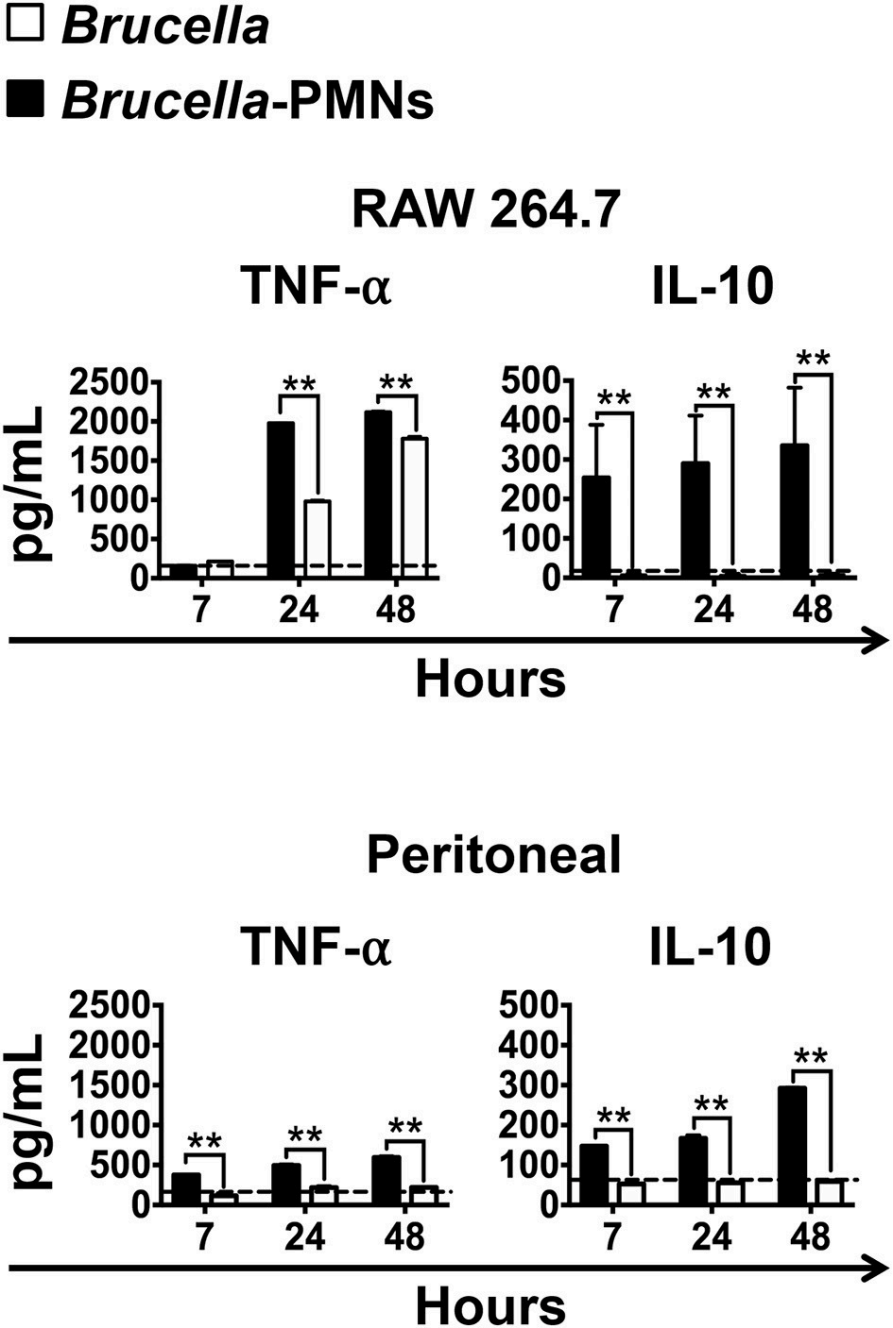
High production of IL-10 and low production of TNF-α in the supernatant of infected Mϕs at initial stages of infection. RAW and peritoneal Mϕs were infected with *B. abortus*-RFP (MOI 50) alone or with *B. abortus-* infected PMNs (MOI 5), and the supernatants collected at different time points. The level of the cytokines was determined by ELISA in the culture supernatants of infected Mϕs. Values of *p* < 0.01 (**) are indicated in relation to Mϕs infected with *Brucella* alone. The backgrounds of the cytokine production by Mϕs co-cultivated with non-infected PMNs are indicated by dashed lines. These experiments were repeated at least three times.

The different bacterial replication kinetic observed between the RAW and the peritoneal Mϕs, seemed related to the distinct profiles of cytokines produced during the infection process (Figure 5). Except for the regulatory IL-10, which was already high (>100 μg), the quantities of the TNF-α were under background levels, at early times of the *Brucella*-PMN infected RAW cells. It is worth noting that RAW Mϕs are TNF-α hyperproducers (24). Therefore, it was expected that at later times, once bacteria reached high numbers, the TNF-α increased to very high levels in the *Brucella-*PMNs infected RAW cells, as compared to the controls. Still, the higher amounts of TNF-α at later times of the RAW infected cells did not hamper bacterial replication. Likewise, at early times of *Brucella*-PMN infection of peritoneal Mϕs, the production of TNF-α was low with significant high amounts of the regulatory cytokine IL-10. These differences in cytokine profiles may explain the differences observed between *Brucella*-PMN-infected RAW and peritoneal Mϕs in the replication kinetics. In any case, in both experiments, *Brucella*-PMN-infected Mϕs reached much higher CFU values than the controls infected with bare bacteria alone.

**Figure 6:**
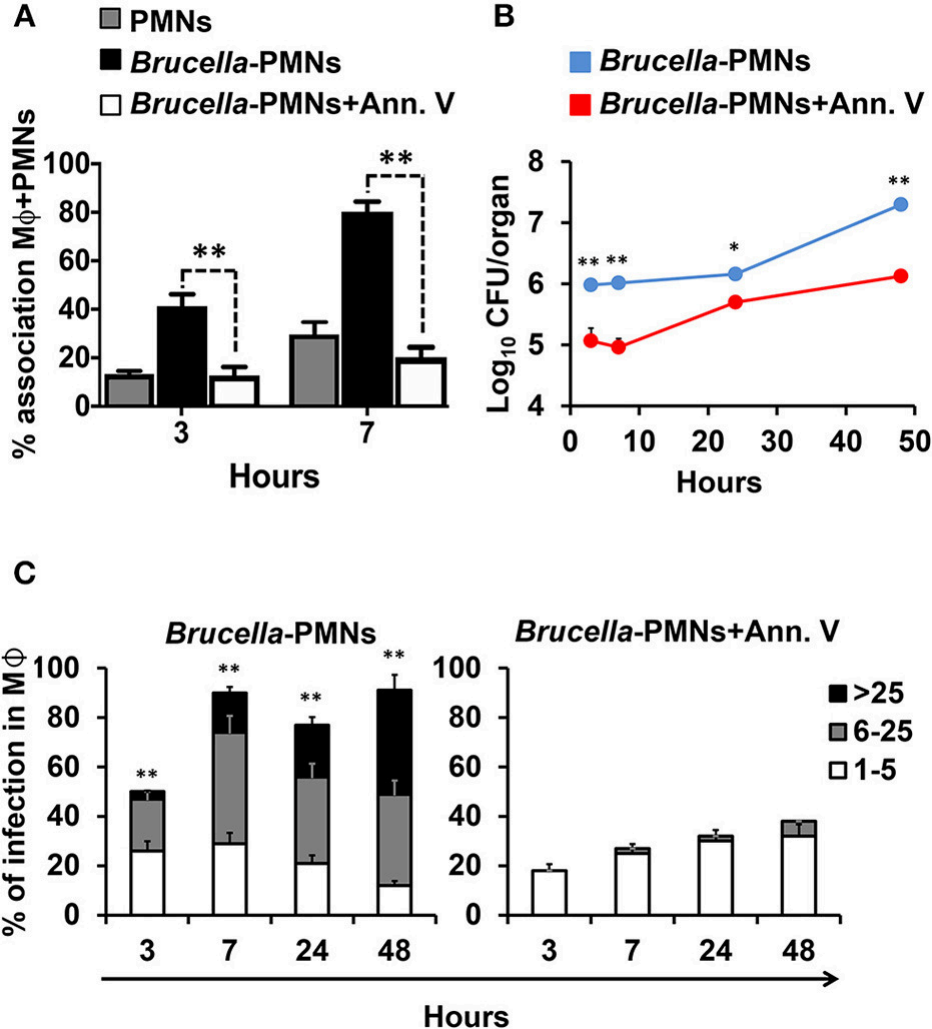
Blockage of PS on the surface of *Brucella-*infected PMNs hampers Mϕ *B. abortus* infection. **(A)** *B. abortus* infected PMNs were pre-treated or not with Annexin V before co-incubation with RAW Mϕs, and cell association estimated. The red dashed line corresponds to the mean value of non-infected PMNs **(B)** *B. abortus* infected PMNs (MOI 5) were pre-treated or not with Annexin V before co-incubation with RAW Mϕs and CFU determined at different time points. **(C)** *B. abortus* infected PMNs were pre-treated or not with Annexin V before co-incubation with RAW Mϕs and the number of infected Mϕs and intracellular bacteria within these cells estimated at different time points. Cells were stained (DAPI for nuclei; phalloidin-FITC for actin filaments), fixed and mounted with MOWIOL. At least 200 cells were counted in each experiment. The percentage of infection and the number of intracellular bacteria (1–5, 6–25, or >25) per Mϕ was determined by fluorescence microscopy. Values of *p* < 0.05 (*) and *p* < 0.01 (**) are indicated in relation to Mϕs incubated with PMNs treated with Annexin V. These experiments were repeated at least three times.

In agreement with our previous reports (13, 21) *Brucella-*infected-PMNs displayed PS on the cell surface (Figure 1B). Since this phospholipid is commonly recognized as an “eat me” signal (14), we decided to explore the role of PS in the uptake of *Brucella-*infected PMNs by Mϕs. For this, we used Annexin V to hinder the PS exposed on the *Brucella*-PMN surface. After treatment with Annexin V, the proportion of *Brucella*-PMNs associated with Mϕs significantly diminished (Figure 6A). Moreover, bacterial replication was reduced in RAW Mϕs at all-time points (Figure 6B), displaying the profile observed after infection with bacteria alone (compare profile with Figure 4A). Likewise, the proportion of infected Mϕs was significantly reduced in the *Brucella*-PMNs treated with Annexin V (Figure 6C). Thus, PS on the *Brucella-*infected-PMNs surface acted as an “eat me” signal for Mϕs.

## Discussion

There are various intracellular pathogens, such as *Chlamydia pneumoniae* and *Leishmania major*, capable to survive within PMNs, kill these cells and use them as vehicles for infecting and colonizing Mϕs (25). This strategy, generally known as the “Trojan horse,” serves as a mechanism for microbial dispersion within the host (15). It seems, therefore, that *B. abortus* also follows a Trojan horse strategy by using infected PMNs as vehicles for the dispersion throughout the host mononuclear phagocytic system. A similar strategy to traverse microvascular endothelial cells of the central nervous system via *B. abortus*-infected-monocytes has been proposed (26).

Infecting naïve Mϕs monolayers (such as bone marrow) with bare *Brucella* grown in a bacteriological medium is highly inefficient (23). Infection protocols in cultured Mϕs require high bacterial MOIs (>50) to obtain low numbers (<5 bacteria/cell) of intracellular bacteria. Moreover, a large proportion of these invading bacteria are killed by Mϕs after a few hours (23). Following this, we propose that the common physiological infection of the phagocytic mononuclear system primarily occurs via *Brucella*-infected-PMNs.

There are at least two other pieces of evidence that support this proposal. First, it has been demonstrated that mice depleted of PMNs, eliminate *B. abortus* more readily than their “normal” infected counterparts (21). This is commensurate with the fact that Mϕs kill bare “unprotected” *Brucella* cells more readily than those hidden within PMNs, as shown here. Second, the early internalization of *Brucella-*infected PMNs by Mϕs, seems to occur in a non-phlogistic manner, displaying significant amounts of regulatory IL-10 and low quantities of proinflammatory cytokines, such as TNF-α at early stages of the infection. It is known, that the uptake of apoptotic PMNs by Mϕs, increases the secretion of anti-inflammatory IL-10 cytokine (27). This is relevant since the first 8 h after cell invasion are crucial for pathogenic *Brucella* to redirect its trafficking to its replicating niche within non-activated cells (23). Indeed, previously activated Mϕs display high brucellicidal activity. However, if Mϕs become activated (e.g., through TNF-α or lipopolysaccharide) after 8–24 h of infection, the intracellular bacteria are still capable to replicate extensively (11). The obvious explanation is that at this infection stage, *Brucella* are hidden within vacuoles of the early phagocytic compartment and then protected from Mϕs microbicidal mechanisms. It is worth noting that the overall activation of the immune system in neutropenic *Brucella* infected mice is considerably higher than in the “normal” infected counterparts indicating that PMNs dampen the adaptive immunity in brucellosis (21, 28).

During the early stages of physiological cell death, PS translocates from the cytoplasmic to the extracellular side of the cell membrane (29). The correct redistribution of PS on the outer surface of the plasmatic membrane is a key element for the recognition of dying cells and corresponds a to molecular “eat me” signal that indicates that these dying cells should be engulfed (30). But PS is also a “forget me” signal for the regulated suppression of Mϕs activating mechanisms (14, 15, 31). Within this context, it seems that ingestion of *Brucella-*infected PMNs by Mϕs follows a similar mechanism used to phagocytize apoptotic PMNs. In any case, it is becoming clearer that through evolution *Brucella* organisms are stealth pathogens that have evolved to hamper the activation of the first stages of innate immunity and to establish chronic infections.

In conclusion, the ability of *Brucella* to circumvent the immune response and to replicate within Mϕs are key elements for the pathogen survival and for the establishing long-lasting infections. Here, we showed that *Brucella*-infected PMNs promoted the internalization and replication of *Brucella* within Mϕs using a “Trojan horse” strategy. To reinforce or reject our hypothesis *in vivo* experiments would be necessary.

In this work our main findings are: (i) *Brucella abortus* infected up to 96% of BM-PMNs, inducing a premature death of these cells; (ii) the *Brucella-*infected PMNs displayed PS as “eat me” signal, promoting the association with Mϕs and favoring the bacterial replication within these mononuclear phagocytes; (iii) This phenomenon was specific, since non-infected PMNs were not phagocytized by Mϕs and blockage of PS with Annexin V diminished the Mϕs association and phagocytosis of *Brucella-*infected PMNs; (iv) the low production of proinflammatory cytokines and the high production of the anti-inflammatory IL-10 at the initial stages of infection, correlated with the non-phlogistic Mϕ *Brucella*-PMN uptake and subsequent bacterial replication.

## Data Availability

The raw data supporting the conclusions of this manuscript will be made available by the authors, without undue reservation, to any qualified researcher.

## Author Contributions

EB-C, EM, and CG-J designed the experiments. CG-J, RM-C, and PA-S performed the experiments. CG-J, RM-C, PA-S, EB-C, EC-O, and EM analyzed the data. CC-D, EC-O, EB-C, and EM contributed reagents, materials, analysis tools. EB-C, EM, and CG-J wrote the paper. All authors revised and approved the manuscript.

### Conflict of Interest Statement

The authors declare that the research was conducted in the absence of any commercial or financial relationships that could be construed as a potential conflict of interest.
